# Retro virology a macro impact in the society

**DOI:** 10.1186/1742-4690-8-S2-P55

**Published:** 2011-10-03

**Authors:** Phillip Nhlanhla

**Affiliations:** 1 University of South Africa, South Africa

## Background

Retrovirology has been widely understood from the biomedical sciences discipline and yet it has got a huge impact in our society. The is a link between the biomedical sciences and the social sciences when coming to the issues of HIV and AIDS which has got both the elements of biomedical science in this regard looking at the retro virology and the social aspects it HIV and AIDS affects everybody and as a results the socio-economic factors of any society in that way a macro impact. The understanding of both the social context and the biomedical context need to be fused together and teach the society to be able to reduce the impact to a smaller scale.

This paper will critically debate the gap between the biomedical sciences and the social science in order to address the huge impact HIV and AIDS has caused within African context. Key results will be drawn from a comprehensive literature review from books and journal articles.

Furthermore, the paper will draw upon some lessons learned and strategic challenges in addressing biomedical and social sciences within the context of HIV and AIDS to make some recommendations and providing guidelines.

